# Supplementary material for: The Sensitivity of Structure to Ionic Radius and Reaction Stoichiometry: A Crystallographic Study of Metal Coordination and Hydrogen Bonding in Barbiturate Complexes of All Five Alkali Metals Li–Cs
Source: Molecules. 2024 Mar 27;29(7):1495. doi: 10.3390/molecules29071495 (PMC11012986; doi:10.3390/molecules29071495)
Supplement: Supplementary file 1 [file molecules-29-01495-s001.zip › molecules-2927780-supplementary.pdf]

The sensitivity of structure to ionic radius and reaction stoichiometry. A crystallographic study of metal coordination and hydrogen bonding in barbiturate complexes of all five alkali metals Li–Cs.

William Clegg and Gary S. Nichol

Supplementary material

Table S1: CSD REFCODES for structures containing BA^- as an uncoordinated discrete anion.

AMBARB01	KEWSIR
BENWUO	KEWSOX
BUYYEA	KINFIZ
BUYYIE	KITGEC
CUHBAL	LUXHAN
CUHBEP	MUYTUW
CUHBOZ	MUYVEI
CUHBUF	MUYVIM
CURKEH	NEBFUY
CURKIL	OQEPAD
ESICOB	OQOXOJ
ESIDES	OQOXUP
ESOZOD	OYANUA
EVELAV	QAFSOF
GATLEU	QEPFIB
GATLIY	QEPFOH
GIGQOD	SUYNIM
HOLFET	UFUWAW
JICWOH10	WAGDIV
JICWOH11	WOPLIY
JURLUG	ZABVAD
KEDZAV	
